# The concept of the digital therapeutic garden and its psychological effects

**DOI:** 10.3389/fpsyg.2025.1534541

**Published:** 2025-10-31

**Authors:** Jisoo Lee, Yeji Yang, Ji-Eun Pyo, Ye-Seul Kim, Kee-Hong Choi, Shin-Koo Kang, Su-Hwan Nam, Yu-Jin Song, Bu-Gi Jeoun, Sung-Hee Park

**Affiliations:** ^1^Department of Psychology, Korea University, Seoul, Republic of Korea; ^2^KU Mind Health Institute, Seoul, Republic of Korea; ^3^Korea Arboreta and Gardens Institute, Sejong, Republic of Korea

**Keywords:** digital healthcare, nature-based therapy, gardening therapy, therapeutic garden, evidence-based intervention

## Abstract

**Introduction:**

Therapeutic gardens have been discussed as effective social interventions for promoting physical and mental health, and integrating digital technologies into therapeutic gardening offers a promising approach to enhance both accessibility and effectiveness. This study aimed to explore various forms of digital therapeutic gardening. Furthermore, it sought to investigate perceptions regarding digital therapeutic gardens and to examine the relationship between participation in such gardens and mental health.

**Methods:**

Survey data were collected from 335 community-dwelling adults in Korea using across-sectional design. Demographic information and experiences with digital gardens were collected, and mental health variables including depression, anxiety, stress, vitality, life satisfaction, loneliness, and social networks were assessed. To investigate differences in mental health by experience with digital therapeutic gardens, *t*-tests and ANCOVA were performed.

**Results:**

Participants with experience in digital therapeutic gardens reported social, psychological, and physical benefits. They also demonstrated significantly higher levels of life satisfaction and vitality than those without such experience.

**Discussion:**

This study highlights the potential therapeutic benefits of digital gardens and suggests that, when integrated with community gardens and mental health services, they may serve as a promising candidate for evidence-based interventions.

## 1 Introduction

Lifestyle-related diseases such as obesity, diabetes, and hypertension have become major public health problems worldwide (Balwan and Kour, 2021). Mental health issues such as depression, isolation, and suicidal ideation have also risen following the COVID-19 pandemic (Bahk et al., 2023; Macalli et al., 2024). Various community-based social interventions have been considered to promote public health, and therapeutic gardening has been utilized as one such intervention in various countries (Spano et al., 2020; Wood et al., 2022). For example, the Eden Project in the United Kingdom offers a gardening program designed to improve mental and physical well-being as a form of social prescription for individuals experiencing health problems in the community (Eden Project, 2024). In Singapore, the National Park Boards select therapeutic garden locations based on proximity to nursing homes and residential areas, accessibility, and natural landscapes, and provide therapeutic garden programs to promote the health and well-being of community residents (National Park Boards, 2024). A therapeutic garden is defined as a deliberately designed, plant-centered environment intended to facilitate interactions with the healing elements of nature (American Horticultural Therapy Association, 2024). Subtypes of therapeutic gardens, including healing, functional, rehabilitation, and restorative gardens, are distinguished from general gardens by their focus on improving physical health rather than spiritual healing and incorporating both horticultural and non-horticultural activities to meet the needs of specific users or patients (Thaneshwari et al., 2018). Therapeutic gardening, a type of nature-based therapy, refers to the use of gardening for therapeutic purposes and involves the non-commercial cultivation, care, and nurturing of plants such as flowers and vegetables (Clatworthy et al., 2013; Soga et al., 2017). Therapeutic gardening activities have been shown to positively affect mental health, reduce negative emotions such as depression, anxiety, and stress, and improve quality of life and subjective well-being (Litt et al., 2023; Park M. O. et al., 2022; Soga et al., 2017). Additionally, research has shown that these activities also benefit physical health and improve social relationships, highlighting their potential as community health and well-being interventions (Fjaestad et al., 2023; Spano et al., 2020; Yang et al., 2023).

Following the coronavirus disease (COVID-19) pandemic, interest in digital healthcare has increased, enhancing the accessibility of therapeutic interventions. The World Health Organization has encouraged the full utilization of digital health interventions to accelerate sustainable health development and universal health coverage, providing classification systems and guidelines (World Health Organization, 2023). Digital health interventions provided through digital technologies such as smartphones, websites, wearable devices, and telemedicine are considered effective, cost-efficient, safe, and expandable (Murray et al., 2016). Considering the advantages of such digital therapeutics, along with the limited opportunities for psychological restoration through direct contact with nature due to social distancing during the COVID-19 pandemic (Mintz et al., 2021), and the difficulty of securing green spaces caused by urbanization and climate change (Derkzen et al., 2017), digital gardens can serve as a highly accessible alternative that provides personalized experiences. In this context, attempts to combine digital technologies such as virtual reality (VR), displays, and mobile applications with therapeutic gardening activities have been growing.

Unlike digital therapeutics, digitizing nature requires careful consideration of the fact that certain environmental benefits inherent to nature–such as sunlight, fresh air, microorganisms, and phytoncides–cannot be replicated digitally. According to Kuo (2015), the mechanisms through which contact with nature promotes human health involve not only environmental factors but also physiological, psychological, and behavioral components. To enhance therapeutic effects, psychological and behavioral elements that can be digitized should be identified and incorporated into digital therapeutic gardening interventions.

In this context, before advancing the research on digital therapeutic gardening activities aimed at promoting mental and physical health, the concept of digital therapeutic gardening and its therapeutic elements must be established. Therefore, this study aims (1) to review existing research on digital therapeutic gardens and discuss their concepts, types, and therapeutic elements, and (2) to empirically examine perceptions, preferences, and participation experiences with digital therapeutic gardens among a community sample in South Korea. By pursuing these aims, we clarify the conceptual framework of digital therapeutic gardening and discuss its theoretical and practical implications, particularly its potential role in enhancing the accessibility and effectiveness of therapeutic gardening interventions.

### 1.1 Concept and types of digital therapeutic gardens

Considering that the incorporation of digital elements in public health can help promote equity (Azzopardi-Muscat and Sørensen, 2019), digital therapeutic gardens can be proposed to enhance accessibility to therapeutic gardens and maximize their effectiveness. Digital therapeutic gardens can be defined as “environments or spaces designed to enhance the therapeutic effects of nature through interactions between natural elements and digital technologies.” Given that therapeutic gardening refers to the use of gardening for therapeutic purposes (Clatworthy et al., 2013), the term digital in digital gardening refers to an digital environment that facilitates the use of nature for therapeutic purposes. Pineda et al. (2023) classified digital mental health interventions (DMHI) into four based on whether a provider administers the intervention and provides therapeutic guidance as followed. Type 1: provider administered DMHIs, type 2: provider administered DMHIs with blended digital, type 3: self-help human supported/guided DMHIs with therapeutic or technical guidance adjuncts, type 4: self-help fully automated DMHIs [For more detailed information, see Pineda et al. (2023)]. According to that, the present study also categorized them into four types: media-based, nature-based, and app-based digital therapeutic gardens and smart gardens. The concepts of each digital type and examples of how digital elements are utilized are explained in the following paragraphs (Table 1).

**TABLE 1:** Examples of digital therapeutic gardens.

Subtype	Author (or name of the case)	Purpose	Contents of digital media	Application and utilization
Media-based	Guided meditation VR	-Therapeutic purpose. -Nature-based meditation	-A nature-based meditation program offering 40 types of natural environment scenes and meditation sessions. -Users can choose from a variety of themes including anxiety, depression, mental fatigue, acceptance, self-compassion, focus, and sleep.	-A meditation timer can be set, allowing users to practice at their preferred time and place. -The program utilizes the Oculus Go VR device, which comes equipped with a built-in headset.
	Heinzerling et al., 2023	-Therapeutic purposes -Combined with hallucinogen-assisted therapy for patients with alcohol use disorder	Through time-lapse, slow-motion, and aerial footage of natural scenes, it offers videos of flowers blooming and fungi sprouting.	-Combined with psychotherapy, it is applied during 2 out of 10 sessions. -Each viewing lasts approximately 40 min, utilizing an 85-inch high-definition LCD television and surround sound speakers.
	Nicholas et al., 2023	Improving the emotional state of patients with delirium	-Dynamic content such as pastoral landscapes, butterflies, and blooming flowers is used. -The video’s dynamism is adjusted through a responsive display that reacts to the patient’s sounds and movements.	-A single 4-h session -Using a 39- or 42-inch HD TV screen, a 720p HD webcam with a built-in microphone, and noise-canceling headphones.
Nature-based	Dipirang, Korea	- Viewing - Strolling	- Features 16 themed zones utilizing light and artificial lighting - Incorporates media art to highlight garden elements such as a sparkling forest, light nets, and a mysterious waterfall	- Covers an area of 128,000 m^2^ - Includes a 1.5 km-long walking path - Open all year round
	Night at the garden, USA	- Viewing - Strolling	- A botanical garden, designed to offer an immersive experience where light and nature converge- Presents a variety of multisensory experiences along its pathways, including rainbow-colored trails, oversized technicolor flowers, and thousands of lights	- Covers an area of 23 acres - Integrating illumination, color, and motion-sensing technology
App-based	Smart home garden	- Prevention of elderly depression and enhanced communication with seniors	- Collects daily data through the app to monitor health status - Tracks plant watering, performance of “positive speech,” app usage time, and daily emotions to support care management - Uses elderly individuals’ app usage time and daily emotions to develop a system that identifies the need for additional attention	Can be utilized at any desired time and place
	Online gardening service, “groo”	- Online community space for gardening and companion plants	- AI diagnoses plant health based on uploaded photos - Offers plant kit purchases - Connects users with professional gardeners - Provides a posting space for companion plant-related content - Includes a plant journal - Enables information sharing with other gardeners	Can be utilized at any desired time and place
Smart garden	Kim and Lee, 2023	- Therapeutic purposes - Mindfulness meditation for inpatients in a day hospital	- Conducts 5-min mindfulness meditation sessions while listening to nature sounds such as streams, birds, and wind, along with the therapist’s guidance voice through speakers - 10 min per session, totaling eight sessions - The first 5 min involve mindfulness meditation, followed by 5 min of relaxation stretching	- Takes place in a cube-shaped indoor garden measuring 3 m wide, 3 m long, and 2.5 m high

### 1.2 Media-based digital therapeutic gardens

Media-based digital therapeutic gardens are those in which the garden environment is implemented using digital media, including VR-based, display-based, and interactive display-based therapeutic gardens. According to classification of Pineda et al. (2023), this type corresponds to provider-administered DMHIs with blended digital adjuncts (Type 2), as the therapeutic effects are delivered through nature imagery administered by providers and mediated via media. VR-based therapeutic gardens provide experiences similar to reality, thereby promoting interactions with nature (Choi et al., 2023). VR has advantages over television and other digital media in that it can induce greater improvements in positive emotions through a vivid sense of presence and natural interactions (Yeo et al., 2020). For example, the “Guided Meditation VR” application provides 40 types of natural environments and meditation options on various topics such as anxiety, depression, mental fatigue, acceptance, self-compassion, concentration, and sleep, and allows users to set a timer. In a study that used this application to offer mindfulness meditation, neurophysiological changes in brain activity were observed, suggesting a reduction in attention to pain during meditation among 10 patients with chronic cancer pain (Fu et al., 2021). Another study comparing the effects of an eight-session VR therapeutic garden program with a control group among elderly women with depressive symptoms found that the group participating in the VR therapeutic garden program showed a 36% reduction in depression scores; this effect persisted in a follow-up assessment (Szczepañska-Gieracha et al., 2021). A study exploring the impact of combining VR digital gardens with horticultural therapy on the physical and mental health of elderly residents in nursing homes found significant improvements in health status, meaning of life, perceptions of importance, loneliness, and depression symptoms in the intervention group compared to the control group, and these improvements were maintained 2 months after the intervention ended (Lin et al., 2020).

Display-based therapeutic gardens involve presenting therapeutic gardens through screens, ranging from small monitors to large media façades. These are typically presented in video format and are often accompanied by sound, with the primary purpose being meditation or mindfulness. Display-based therapeutic gardens can feature videos of actual gardens or forests or can be presented as media art using large media façades. A common example is mindfulness or meditation videos featuring gardens or natural plant scenes that can be easily found on YouTube. However, there is a lack of rigorous testing of the therapeutic effects of this type of treatment, and further randomized controlled trials are needed to verify its efficacy.

Interactive display-based therapeutic gardens involve the attachment of cameras and microphones to the display, allowing appropriate visual presentation based on the movements and sounds of the user, thereby enhancing interaction with nature compared with traditional display-based therapeutic gardens. Nicholas et al. (2023) exposed patients with delirium to a digital mindfulness garden via a display for 4 h, resulting in a significant reduction in agitation scores compared to the control group. The display, positioned at the foot of the patient’s bed, showed scenes of pastures, butterflies, and blooming flowers with a camera and microphone attached to the display, adjusting the dynamics of the video based on the patient’s sound and movement.

### 1.3 Nature-based digital therapeutic gardens

A nature-based digital therapeutic garden aims to enhance the interactive effects of natural elements by incorporating digital media within an actual therapeutic garden. It utilizes lighting, video, sound, and other sensory stimulus to create multisensory landscapes within preexisting parks or natural spaces. This type, as described by Pineda et al. (2023), falls under Type 4, which involves self-help fully automated DMHIs. By establishing digital technology–integrated therapeutic gardens in advance, participants can access them at any time according to their needs. Tongyeong Dipirang, a digital sculpture park in Korea, combines natural landscapes with light and video installations, enhancing visitors’ sense of presence and immersion (Park, 2023). Exposure to various natural elements and garden spaces supports participants in experiencing diverse sensory stimuli, including visual and auditory, while fostering a sense of control (Singapore National Parks Board, 2025). In Nagai Park, Osaka, digital elements highlight natural features and reconfigure the garden environment. An indoor space presents interactive installations where visitors engage with the artwork, illustrating the interconnectedness of humans and nature within the ecosystem (TeamLab, 2022). The Light Art Festival “Island of Light,” located on the western coast of Sweden, offers a unique experience that fosters a sense of connection with nature (Island of Light, 2018). The Night at the Garden in Florida, USA, is a botanical garden spanning 23 acres, designed to offer an immersive experience where light and nature converge. By integrating illumination, color, and motion-sensing technology, the garden presents a variety of multisensory experiences along its pathways, including rainbow-colored trails, oversized technicolor flowers, and thousands of lights (Cahill, 2024). Other examples of digital art integration within gardens include the Morning Calm Arboretum in Korea, the Royal Botanic Gardens in Melbourne, Tokyo Digital Art Garden, and Rakuten Garden.

Nonetheless, research on the physical and mental health benefits of nature-based digital therapeutic gardens is limited. However, studies have reported air quality improvement effects of vegetation within garden environments (Kuo, 2015) and the antimicrobial effects of phytoncides, and organic compounds emitted by plants (Li et al., 2009, 2011). Additionally, the visual and auditory stimuli of natural landscapes have been found to positively influence the immune system by increasing parasympathetic nervous system activity (Alvarsson et al., 2010; Gladwell et al., 2012; Kenney and Ganta, 2014). Although more rigorous studies are needed to validate these effects, the use of digital media such as light, video, and mindfulness guides in conjunction with natural sensory stimuli may potentially enhance the therapeutic efficacy of these environments.

### 1.4 App-based digital therapeutic gardens

App-based digital therapeutic gardens are characterized by enabling interaction with therapeutic gardens through applications that utilize Internet of Things (IoT) technology. Users can interact with a garden system without being constrained by time or space, thereby enhancing their engagement with nature and providing opportunities for social interaction through the application. This type can be classified as Self-help human supported/guided DMHIs with therapeutic or technical guidance (type 3; Pineda et al., 2023). Although users engage with the application autonomously, difficulties in its use are addressed and sustained participation is encouraged by a facilitator. These advantages are particularly beneficial for individuals with limited mobility, such as older adults, as they promote sustained interactions with natural elements and other people. For instance, an IoT-based smart home garden application has been developed to prevent depression and promote communication among the elderly (Lee et al., 2022). It utilizes soil moisture sensors, temperature and humidity sensors, and app-based records to collect daily data, such as app usage time, daily mood records, and plant watering schedules, thereby monitoring the user’s health status and providing connections to social welfare services when needed. This type of non-face-to-face welfare system is expected to enhance the efficiency of welfare and mental health promotion among older adults.

Similarly, in the Turntable Solution Project (Vassányi et al., 2022), applications such as “Tomappo,” which allows older adults to collect plant-related information and design and record garden layouts, and “Lifely,” which integrates with light, temperature, and soil moisture sensors and a mini water pump, were used to support participants’ gardening activities. After 3 months of using these applications for gardening, participants showed significant improvements in their quality of life and cognitive function, as well as a non-significant trend toward reduction in depression and loneliness. Moreover, this approach is advantageous not only for promoting mental health through gardening activities but also for enabling the observation of moment-to-moment changes in participants’ mental and physical states by recording their condition through the application.

### 1.5 Smart gardens

A smart garden is a new type of garden that consists of indoor plants planted in modular units equipped with automated lighting and irrigation systems. This type is considered Provider administered DMHIs (Type 1; Pineda et al., 2023). Smart garden is maintained and managed by facilitators, and within smart garden, providers deliver interventions such as counseling or meditation. It is categorized into three types based on its structure: cube-shaped, built as a rectangular booth; wall-mounted, with plants planted on vertical surfaces; and a hybrid model combining both (Korea Arboretum and Garden Management Service, 2024). Smart gardens utilize IoT technology to introduce automated plant management systems that minimize human intervention, thereby reducing maintenance costs.

In a study that examined the effects of a mindfulness meditation program conducted within a smart garden among patients in a daycare hospital, participants showed a significant reduction in depression and anxiety, whereas their quality of life showed a positive trend, although this was not statistically significant (Kim and Lee, 2023). Physical health and parasympathetic nervous system function also improved, which the authors interpreted as a smart garden program promoting physiological stability by activating the parasympathetic nervous system. In that study, the smart garden was designed as an indoor garden in a cubic shape, measuring 3 m in width, 3 m in length, and 2.5 m in height, with lighting installed within and one side made of glass. The mindfulness meditation program involved listening to recorded natural sounds and therapist-guided instructions, followed by three repetitions of naming objects, taking deep breaths, and concluding with 5 min of muscle relaxation stretching.

### 1.6 Therapeutic elements of digital therapeutic gardens

A review of previous studies suggests that the following three elements are essential to enhance the therapeutic effects of digital therapeutic gardens: a sense of connection to nature and presence, active participation within the garden space, and mindfulness. The first component is a sense of connection between nature and presence, which refers to the feeling of being immersed in a therapeutic garden environment and connected to nature. Connectedness with nature pertains to an individual’s emotional, cognitive, and experiential relationship with the natural world or their subjective connection with nature (Nisbet et al., 2011). In nature-based interventions, connectedness with nature has been associated with psychological, emotional, and general well-being as well as life satisfaction (Wu and Jones, 2022). In VR-based therapy, the sense of presence, which enhances immersion in virtual reality, is considered a critical factor and can be defined as the “subjective experience of being in one place or environment, even when one is physically situated in another” (Witmer and Singer, 1998, p. 1). In virtual reality experiences that simulate natural environments, a high level of presence and connectedness with nature are key factors that influence improvements in positive emotions (Yeo et al., 2020). The sense of presence has also been shown to have a significant impact on therapeutic outcomes in VR-based psychotherapy (Augustin et al., 2024). Similarly, presenting dynamic and colorful animations, rather than static environments, can increase immersion in digital therapeutic gardens (Nicholas et al., 2023; Szczepañska-Gieracha et al., 2021). Thus, the degree of immersion, including a sense of presence and connectedness with nature, is considered critical in enhancing therapeutic effects when designing digital garden therapeutic environments.

Active participation within the garden space involves actively participating in the therapeutic garden. A primary characteristic of therapeutic gardens is the presence of immersive activities and physical engagement (Hazen, 2012). Gardening activities, which include more direct interaction with nature in a goal-oriented manner, have been found to be more effective for stress recovery compared to activities such as observing nature or walking in natural environments (Van Den Berg and Custers, 2011). Additionally, previous research on VR natural environments has shown that VR-based nature experiences are more effective in improving positive emotions than videos, including high-definition and 360-degree videos (Yeo et al., 2020). Therapeutic effects are further enhanced when active interactions such as watering plants or fishing are included within the virtual therapeutic garden space during the VR experience (Li H. et al., 2021; Li et al., 2022). This suggests that engaging in active experiences rather than passively existing in the environment can induce stronger therapeutic effects in digital therapeutic gardens.

The third component, mindfulness, refers to deliberately and attentively recognizing one’s internal state and surroundings (American Psychological Association, 2018). Mindfulness helps individuals avoid habitual or destructive behaviors and reactions by teaching them how to observe their thoughts, emotions, and present experiences without judging or reacting. When experiencing a therapeutic garden with mindfulness, individuals can intentionally and attentively focus on the moment and immerse themselves in nature without judgment. Ulrich’s (1983) Stress Reduction Theory (SRT) emphasizes that exposure to natural settings reduces physiological stress responses and promotes positive affect through activation of the parasympathetic nervous system. According to Kaplan and Kaplan’s (1989) Attention Restoration Theory, exposure to nature promotes recovery because natural environments are physically distanced from the stressors of daily life and because nature fosters “soft fascination,” which refers to the effortless attention drawn to captivating objects. Together, these theories highlight that natural environments not only induce immediate stress relief but also restore depleted attentional resources. Mindfulness cultivates this capacity, enabling individuals to sustain awareness of the natural environment and thereby maximize both the restorative and stress-reducing effects described in ART and SRT. Natural environments are sufficiently expansive and rich, have coherent patterns that facilitate stress relief, and easily engage attention (Djernis et al., 2019). To achieve the therapeutic effects of natural exposure, it is necessary to intentionally direct attention to the natural environment (Jiang et al., 2019). Numerous studies have demonstrated that nature-based mindfulness activities are effective in psychological, physiological, and social aspects (Djernis et al., 2019), and mindfulness has been identified as a key mediator of the effect of therapeutic gardening activities on reducing depression and anxiety and improving life satisfaction (Kang et al., 2023). Therefore, mindfulness, which involves directing intentional attention toward the present experience, is a crucial element in eliciting sufficient therapeutic effects from the digital environment.

This study aims to explore various forms of digital therapeutic gardens and conduct a community survey on perceptions, preferences, and participation experiences regarding digital therapeutic gardens. Based on previous research, this study proposes the following hypotheses. First, individuals who experience a sense of connectedness with nature, active participation, and mindfulness within digital therapeutic gardens are expected to report psychological therapeutic effects, as discussed in prior studies. Furthermore, they are expected to report on the physical therapeutic effects and social benefits of gardening activities and social interactions. Second, individuals with participation experience in digital therapeutic gardens are expected to exhibit better mental health outcomes, such as lower depression and anxiety, and higher quality of life, compared to those without such experiences.

## 2 Materials and methods

### 2.1 Research design and procedure

This study collected data cross-sectionally from a Korean community population through an online survey. All data collection procedures were conducted independently of the research team by the professional research agency, OLIM. Participants who indicated willingness to participate in the study received a unique survey link generated through the company’s proprietary software, V3. Prior to participation, informed consent was obtained from all respondents. To ensure data quality, response times and atypical response patterns were examined, and only participants who provided valid and diligent responses were compensated with KRW 5,000. The dataset was fully anonymized to remove any personal identifiers, and access was restricted to the research team. Participants were divided into groups based on whether they had experienced a digital therapeutic garden or not. Perceptions of the digital therapeutic garden and psychological factors - depression, anxiety, stress, vitality, satisfaction with life, loneliness and social network - were compared between the groups. The study framework is presented in Supplementary Figure 1. All the study procedure were approved by the Institutional Review Board of Korea University (KUIRB-2024-0049-02).

### 2.2 Participants

Data was collected via an online survey in May 2024. Stratified sampling was employed based on environmental and demographic characteristics to ensure regional representativeness of Korea. South Korea is administratively divided into the capital city, Seoul, and six provinces (“do”). Considering that approximately half of the country’s population resides in the Seoul metropolitan area, including Seoul and Gyeonggi Province, around 50% of the responses were collected from this region, while the remaining responses were gathered in accordance with the population distribution across the other regions. The inclusion criteria were as follows: (1) adults aged 19 years or older, and (2) individuals proficient in Korean, able to understand the survey without difficulty. In total, data were collected from 355 participants.

### 2.3 Measures

#### 2.3.1 Demographic variables

Demographic information, including gender, age, place of residence, socioeconomic status (educational attainment and employment status), marital status, type of residence, and household size, was collected. The age of the participants was determined by their date of birth, and educational attainment was measured based on the highest completed level of education. Household size included participants and cohabitants. Differences in digital therapeutic garden experiences were examined across demographic variables.

#### 2.3.2 Experience with digital therapeutic gardens

A survey was conducted with participants who had experience with digital therapeutic gardens and those who had not. The questionnaire covered participation status, intention to participate, preferred digital garden type, purpose of participation, and expected therapeutic effects. Definitions and examples are provided for clarity. Examples of nature-based digital therapeutic gardens include the Suncheonman National Garden Media Art and the Garden of Morning Calm in Gapyeong. Media-based digital therapeutic gardens include a high-definition digital mindfulness garden (Nicholas et al., 2023) and a VR-based therapeutic garden (Szczepañska-Gieracha et al., 2021). App-based digital therapeutic gardens and smart gardens were represented by the smart garden model of Kim and Lee (2023).

To examine the psychological, physical, and social therapeutic effects of digital gardens and their relationship with mental health and physical activity levels, items were adapted from the Gardner Benefits Questionnaire (Scott et al., 2020), with additional items developed for the purpose of the study. Multiple responses were provided regarding the type of digital therapeutic garden experienced, future participation intentions, preferences, and expected therapeutic effects. This approach aimed to provide a comprehensive analysis of the various types and therapeutic effects of digital gardens and offered foundational data for developing effective models in the future. The results are presented in Table 2.

**TABLE 2:** Questionnaire of experiences with digital therapeutic gardens.

Category	Number of questions	Questions
Demographic variables	8	① What is your age? ② What is your gender? ③ What is your current place of residence? ④ What is the highest level of education you have completed? ⑤ What is your current employment status? ⑥ What is your current marital status? ⑦ What is your current type of residence? ⑧ How many people, including yourself, live in your household?
**Participation experience**		
Participation intent	3	① How willing are you to participate in nature-based therapeutic gardens? ② How willing are you to participate in media-based therapeutic gardens? ③ How willing are you to participate in app-based therapeutic gardens or smart gardens?
Therapeutic effects	3	① To what extent have you experienced psychological therapeutic effects from participating in digital therapeutic gardens? (e.g., “I feel calm after visiting the garden,” “The garden helps me manage stress.”) ② To what extent have you experienced physical therapeutic effects from participating in digital therapeutic gardens? (e.g., “I feel more energetic through gardening activities,” “Physical activities in the garden have improved my strength.”) ③ To what extent have you experienced social therapeutic effects from participating in digital therapeutic gardens? (e.g., “Meeting new friends through gardening activities has been helpful,” “The garden provides a space for communication with others.”)
Reason for participation	1	① What was the main reason for your visit to the digital therapeutic garden?
Satisfaction	1	① How satisfied are you with your experience in the digital therapeutic garden?
**No participation experience**		
Participation preferences	3	① How willing would you be to visit a nature-based therapeutic garden? ② How willing would you be to visit a media-based therapeutic garden? ③ How willing would you be to visit an app-based therapeutic gardens or smart gardens?
Reasons for non-participation	1	① What are the reasons you have not participated in a digital therapeutic garden?
Expected therapeutic effects	1	① If you visit a digital therapeutic garden, what psychological therapeutic effects do you expect? (e.g., “I expect to feel calm after visiting the garden,” “The garden will help me manage stress.”) ② If you visit a digital therapeutic garden, what physical therapeutic effects do you expect? (e.g., “I expect to feel more energetic through gardening activities,” “Physical activities will improve my strength.”) ③ If you visit a digital therapeutic garden, what social therapeutic effects do you expect? (e.g., “Meeting new people will be helpful,” “The garden will provide opportunities to meet and engage with others.”)

#### 2.3.3 Depression

The Mental Health Screening Tool for Depression-2 is a brief self-report measure for Korean developed by Park K. et al. (2022) with high accuracy for the early detection of major depressive disorder in primary care settings. It consists of two items from the first and second questions of the original scale, using a 5-point Likert scale ranging from “not at all (0)” to “very much (4).” The total score ranges from 0 to 8, with each item reflecting a core diagnostic domain of major depressive disorder: depressed mood and loss of interest. A validation study was conducted in South Korea (Choi et al., 2022), demonstrating high internal consistency (Cronbach’s α = 0.89). The internal consistency analyzed based on our data was also acceptable (Cronbach’s α = 0.88).

#### 2.3.4 Anxiety

The Mental Health Screening Tool for Anxiety-2 is a brief self-report measure for Korean developed by Kim et al. (2021) for early detection of generalized anxiety disorder in primary care settings with high accuracy. It consists of two items from the second and ninth questions of the original scale and uses a 5-point Likert scale ranging from “not at all (0)” to “always (4).” The total score ranges from 0 to 8, with each item reflecting a core diagnostic domain of generalized anxiety disorder: difficulty in controlling worry and functional impairment due to anxiety. It was validated in South Korea (Choi et al., 2022), with the scale demonstrating good internal consistency (Cronbach’s α = 0.80). The internal consistency based on our data was also acceptable (Cronbach’s α = 0.83).

#### 2.3.5 Stress

The Korean version of the Perceived Stress Scale is an adapted version of the original scale developed by Cohen et al. (1983) for assessing perceived stress. It consists of 10 items using a 5-point Likert scale ranging from “never (0)” to “very often (4),” based on the participant’s stress experiences over the past month. The total score ranged from 0 to 40, with higher scores indicating greater perceived stress. A domestic validation study has reported good internal consistency (Cronbach’s α = 0.81; Lee et al., 2012).

#### 2.3.6 Core life activities

The Core Life Activities (CORE) scale developed by Cho et al. (2024) measures an individual’s engagement in five key areas over the past week: sleep, eating, physical activity, intimate relationships, and learning new things. The CORE scale was used to assess vitality. It consists of five items rated on a 5-point Likert scale ranging from “not at all (1)” to “very much (5),” based on participants’ experiences during the past week. A validation study in Korea reported good internal consistency (Cronbach’s α = 0.77; Cho et al., 2024).

#### 2.3.7 Satisfaction with life

The Satisfaction with Life Scale is a self-report measure developed by Diener et al. (1985) and translated and validated in Korean by Cho and Cha (1998). It consists of five items that measure life satisfaction, which is an aspect of subjective well-being. Each item is rated on a 7-point Likert scale ranging from “strongly disagree (1)” to “strongly agree (7),” with total scores ranging from 5 to 35. A domestic validation study by Lim (2012) reported high internal consistency (Cronbach’s α = 0.84–0.91).

#### 2.3.8 Loneliness

The Short Form of the UCLA Loneliness Scale-8 (UCLA-8) is a brief version of the original scale developed by Russell et al. (1978) to measure loneliness and is reduced to eight items (Hays and DiMatteo, 1987). The UCLA Loneliness Scale is one of the most widely used measures for assessing loneliness, and a Korean validation study reported high internal consistency (Cronbach’s α = 0.93). The UCLA-8 was created by condensing the original 20 items into 8 to improve ease of use and demonstrated strong internal consistency at the time of development (Cronbach’s α = 0.84; Jin and Hwang, 2019).

#### 2.3.9 Social network

The Lubben Social Network Scale (LSNS) was developed by Lubben (1988) to assess the social networks of older adults and translated into Korean to evaluate its reliability and validity (Kang, 2011). This study used the 6-item short form of the LSNS (LSNS-6; Lubben and Gironda, 2000). Each item is rated on a 5-point Likert scale ranging from “none (0)” to “9 or more (4),” with a total score ranging from 0 to 30. Lower scores indicate greater social isolation. A domestic study reported good internal consistency (Cronbach’s α = 0.83; Hwang et al., 2021).

### 2.4 Statistical analysis

Statistical analyses were conducted using Statistical Package for the Social Sciences (SPSS version 27.0). First, frequency analyses were conducted to examine the participation rates in digital therapeutic gardens based on demographic characteristics and garden type. For participants with experience, frequency analyses included garden type, future participation intentions, subjective satisfaction, and perceived therapeutic effects. For those without such experience, the analyses included reasons for non-participation, future participation intentions and preferences, and expected therapeutic effects according to garden type. Second, we utilized skewness, kurtosis values, and Q-Q plots for normality assumption checking, followed by independent sample *t*-tests to compare the levels of depression, anxiety, stress, vitality, loneliness, and social networks between participants with and without experience in digital therapeutic gardens. Additional independent sample *t*-tests were conducted to compare stress, vitality, and loneliness based on participation status and whether depression or anxiety scores exceeded the cutoff threshold. Third, a two-way analysis of covariance (ANCOVA) was conducted to examine the effects of depression and anxiety cutoff levels and participation in the digital therapeutic garden on mental health outcomes By using two-way ANCOVA, we simultaneously tested the main and interaction effects while controlling for demographic covariates (gender, age, residence, residence type, job status, and marital status) that may influence mental health.

## 3 Results

### 3.1 Descriptive characteristics

This study included 335 participants, 47.8% of whom were male and 52.2% were female. The age distribution showed that participants aged 60 years and older constituted the largest group (31.6%), followed by those in their 50s (20%), 40s (17.3%), 30s (16.1%), and 20s (14.9%). The key demographic characteristics of the participants are presented in Table 3.

**TABLE 3:** Demographics.

Characteristics		*n*, %
Gender	Male	160 (47.8)
	Female	175 (52.2)
Age	20s	50 (14.9)
	30s	54 (16.1)
	40s	58 (17.3)
	50s	67 (20.0)
	60s and over	106 (31.6)
Residence (province)	Seoul/Gyeonggi	162 (48.4)
	Chungcheong-do	39 (11.6)
	Joella-do/Jeju-do	42 (12.5)
	Gyeongsang-do/Gangwon	92 (27.5)
Residence type	Detached house	33 (9.9)
	Apartment	249 (74.3)
	Townhouse/multi-unit house	48 (14.3)
	Others	5 (1.5)
Job	Full time	170 (50.7)
	Part time	41 (12.2)
	Jobless	28 (8.4)
	Student	15 (4.5)
	Retirement	22 (6.6)
	Housewife	59 (17.6)
Marital status	Unmarried	116 (34.6)
	Married	197 (58.8)
	Divorced	17 (5.1)
	Bereavement	5 (1.5)

### 3.2 The perception and preferences of digital therapeutic gardens

A frequency analysis of participation showed that out of 335 participants, 113 (34%) had experience with digital therapeutic gardens. By contrast, 222 (66%) did not. The most frequently experienced type was nature-based digital therapeutic gardens (46%). The type participants were most willing to try was the media-based digital therapeutic garden (79%). The primary reasons for participating were garden appreciation (43%), relaxation (35%), walking (11%), meetings and conversations (5%), and exercise (1%). Additionally, 83% of the participants reported satisfaction with their digital therapeutic garden experience.

In terms of perceived healing effects, participants reported experiencing social interaction benefits (e.g., “It helps me meet new people,” “I have met or maintained friendships through gardening activities”), psychological benefits (e.g., “When I am stressed, I feel at ease when I go to the garden,” “I feel good when I go to the garden”), and physical benefits (e.g., “My physical activity level increases and my body feels more energetic,” “It can prevent diseases”), with experience rates of 81%, 60%, and 44%, respectively.

For those who had not participated, the main reasons were “difficulty using digital devices and technical challenges” (79%), “lack of interest in” (17%), and “lack of information about digital therapeutic gardens” (4%). The interest in future participation was positive. The preferred types were nature-based (69%), app-based and smart gardens (56%), and media-based digital therapeutic gardens (47%). The expected therapeutic effects for future participation included psychological effects (93%), physical effects (89%), and social interaction and relationship enhancement (86%). Table 4 presents the survey findings on digital therapeutic garden awareness based on the participation status.

**TABLE 4:** Survey on awareness of digital healing gardens (*N* = 335).

Survey question	Digital healing garden types			
		Nature based	Media based	App-based and smart garden
**Participated (*n* = 113, 34%)**				
Participated		67 (46%)	49 (34%)	29 (20%)
Willingness to participate		86 (77%)	89 (79%)	73 (72%)
Therapeutic effects experienced	Psychological		68 (60%)	
	Physical		50 (44%)	
	Social Interaction		92 (81%)	
Purpose of participation	Gardening		48 (43%)	
	Relaxation		40 (35%)	
	Walking		12 (11%)	
	Meeting		6 (5%)	
	Conversation		6 (5%)	
	Exercise		1 (1%)	
Satisfaction level			94 (83%)	
**No participation (*n* = 222, 66%)**				
Willingness to participate		133 (68%)		
Participation preference		136 (69%)	33 (47%)	109 (56%)
Reasons for non-participation	Difficulty with digital devices and technical challenges		175 (79%)	
	Lack of interest in digital therapeutic gardens		38 (17%)	
	Lack of information on digital therapeutic gardens		9 (4%)	
Expected level of therapeutic effects	Psychological		206 (93%)	
	Physical		197 (89%)	
	Social interaction		190 (86%)	

### 3.3 Difference in mental health based on experience in a digital therapeutic garden

Prior to conducting the independent sample *t*-tests, normality was examined. The skewness and kurtosis values were within acceptable ranges (| 3| and | 7|, respectively; Kline, 2005), and the Q-Q plots indicated approximate linearity, supporting the assumption of normality. Then we explored the impact of digital therapeutic gardens on various mental health variables. It found no significant differences in depression, stress, vitality, or loneliness between the groups that participated in digital therapeutic gardens and those that did not. However, there were significant differences in anxiety (*t* = 2.097, *p* = 0.037), life satisfaction (*t* = 2.873, *p* = 0.004), and social networks (*t* = 3.021, *p* = 0.003) between the groups. Specifically, the group that participated in the digital therapeutic gardens reported significantly higher levels of anxiety, life satisfaction, and social network connections than the non-participating group. Table 5 presents detailed results.

**TABLE 5:** Group differences in the major variables.

Variables	Participated (*n* = 113)	No participation (*n* = 222)	*t*
	*M* (SD)	*M* (SD)	
Depression	2.60 (2.21)	2.21 (1.90)	1.61
Anxiety	2.40 (2.37)	1.85 (2.00)	2.09*
Stress	18.21 (4.72)	18.13 (5.56)	0.14
Life satisfaction	19.63 (6.36)	17.51 (6.39)	2.87**
Vitality	15.81 (3.18)	15.37 (3.26)	2.00
Loneliness	15.07 (4.36)	14.84 (4.56)	0.44
Social network	20.36 (5.52)	18.35 (5.90)	3.02**

*p < 0.05;

**p < 0.01.

**FIGURE 1:**
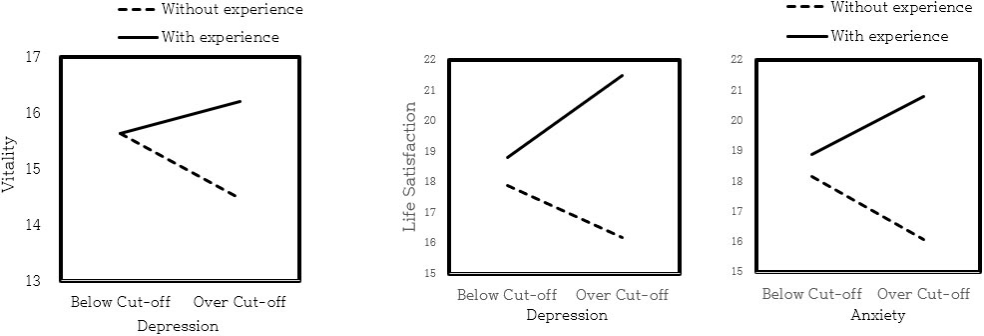
Impact of digital therapeutic garden participation on mental health (vitality and life satisfaction) by depression and anxiety levels (ANCOVA).

We conducted a two-way ANCOVA to examine the interaction effect of depression and anxiety levels alongside digital participation experience on mental health outcomes, while controlling demographic covariates (gender, age, residence, residence type, job status, and marital status). None of these covariates showed significant effects. The results indicated a significant interaction between the depression level and participation in digital therapeutic gardens for vitality (*F* = 4.789, *p* = 0.029), and life satisfaction (*F* = 7.531, *p* = 0.006). Additionally, we observed a significant interaction between anxiety levels and participation in digital therapeutic gardens on life satisfaction (*F* = 6.796, *p* = 0.010). Specifically, in the group with depression levels above the cutoff, those with experience reported higher vitality, and greater life satisfaction than those without such an experience. Similarly, for individuals with anxiety above the cutoff, those with digital therapeutic garden participation showed higher life satisfaction than those without this experience. Figure 1 and Table 6 present these findings.

**TABLE 6:** A two-way analysis of covariance (ANCOVA) of mental health variables (vitality and life satisfaction) by depression and anxiety level and participation in the digital therapeutic garden.

Variables	Source	Degrees of freedom	Sum of squares	Mean square	F	η ^2^
Vitality	Depression level (A)	1	6.74	6.74	0.65	0.00
	Experience of digital therapeutic garden participation (B)	1	42.35	42.35	4.05*	0.01
	A × B	1	50.09	50.09	4.79*	0.02
	Gender	1	1.97	1.97	0.19	0.00
	Age	1	1.14	1.14	0.11	0.00
	Residence	1	2.37	2.37	0.23	0.00
	Residence type	1	7.75	7.75	0.74	0.00
	Job	1	1.00	1.00	0.10	0.00
	Marital status	1	4.58	4.58	0.44	0.00
	Error	325	3399.44	10.46		
	Total	335	84187.00			
Life satisfaction	Depression level (A)	1	13.18	13.18	0.33	0.00
	Experience of digital therapeutic garden participation (B)	1	533.94	533.94	13.28***	0.04
	A × B	1	302.68	302.68	7.53**	0.02
	Gender	1	17.76	17.76	0.44	0.00
	Age	1	83.36	83.36	2.07	0.01
	Residence	1	45.63	45.63	1.14	0.00
	Residence type	1	64.62	64.62	1.61	0.01
	Job	1	13.51	13.51	0.34	0.00
	Marital status	1	27.88	27.88	0.69	0.00
	Error	325	13062.97	40.19		
	Total	335	125165.00			
	Anxiety level (A)	1	1.38	1.38	0.03	0.00
	Experience of digital therapeutic garden participation (B)	1	460.51	460.51	11.46***	0.03
	A × B	1	273.04	273.04	6.80*	0.02
	Gender	1	20.16	20.16	0.50	0.00
	Age	1	78.78	78.78	1.96	0.01
	Residence	1	34.62	34.62	0.86	0.00
	Residence type	1	51.46	51.46	1.28	0.00
	Job	1	19.92	19.92	0.50	0.00
	Marital status	1	18.55	18.55	0.46	0.00
	Error	325	13056.84	40.18		
	Total	335	125165.00			

*p < 0.05,

**p < 0.01,

***p < 0.001.

## 4 Discussion

### 4.1 Main findings

This study reviewed the existing research on digital therapeutic gardens, established their concepts and types, and suggested therapeutic elements for digital garden activities, considering the therapeutic factors of nature-based gardening and the characteristics of digital media. Furthermore, this study examined the perceptions and preferences of community dwellers and explored the relationship between digital therapeutic gardens, their therapeutic effects, and mental health.

Digital therapeutic gardens utilizing digital media to promote interaction with nature and fostering healing effects were categorized into four types: media-based (e.g., VR, media art), nature-based, and app-based digital therapeutic gardens and smart gardens. Upon reviewing existing research, three therapeutic elements for digital gardens are suggested: (1) a sense of connection to nature and presence, (2) active participation within the garden space, and (3) mindfulness. These elements are considered to promote participants’ psychological, physical, and social therapeutic effects (Djernis et al., 2019; Soga et al., 2017) and thus should be considered as key variables in the construction of digital therapeutic gardens.

Based on the frequency analysis of participation, digital therapeutic gardens have reached a moderate level of engagement among the study population, with 34% of participants reporting prior experience. Nature-based digital therapeutic gardens were the most experienced type, yet media-based gardens were the most appealing for future use, suggesting a potential shift in user preference toward more interactive or technology-mediated formats. It indicates positive user experiences that participants primarily engaged in these gardens for appreciation of the environment and relaxation, and a high satisfaction rate (83%). Regarding perceived therapeutic effects, social interaction benefits were reported most frequently (81%), followed by psychological (60%) and physical benefits (44%), highlighting the various values of these interventions. Among participants without experience, the predominant barriers were technical challenges and difficulties using digital devices, indicating that accessibility remains a critical factor. Nevertheless, interest in future participation was high, with participants expecting psychological, physical, and social benefits, particularly from nature-based and app- or media-based gardens. These findings underscore both the promise of digital therapeutic gardens in promoting well-being and the importance of addressing usability and informational barriers to expand engagement. Notably, the participants reported a pronounced impact on social interactions, contrary to the expectation that they would primarily report psychological effects. This result aligns with previous studies indicating that therapeutic gardens promote social interaction and bonding (Spano et al., 2020), suggesting that digital therapeutic gardens can enhance social interaction. This might be explained by the Theory of Interactive Media Effect (TIME; Sundar et al., 2015). The TIME model suggests that Human to Human and Human to Text interactivity increases the social presence in digital environment, which leads to user’s satisfaction in digital social reading (Li W. et al., 2021). Their potential to address isolation and loneliness through improved social interaction is worth exploring. Future studies should focus on the effectiveness of digital therapeutic gardens by considering improvements in social interactions as a key variable.

Analysis of the relationship between participation in digital therapeutic gardens and mental health variables showed that individuals with prior experience reported significantly higher life satisfaction and vitality, confirming the benefits of therapeutic gardens. Notably, there was no significant difference in depression scores between the groups based on participation experience, and in the case of anxiety, those with participation experience reported higher anxiety, contrary to the initial hypothesis. This result could be reported because the participants usually expect to decrease stress level through therapeutic gardens (Lee and Yeon, 2021), so people with higher stress would be more inclined to take part in therapeutic gardens. It is necessary to figure out the causal relationship by RCT or longitudinal research. Further analysis revealed that in groups with depression levels above the cutoff, individuals with experience showed higher vitality, life satisfaction, and lower stress. Similarly, in groups with anxiety levels above the cutoff, individuals with experience reported higher life satisfaction than those without. Thus, participation in digital therapeutic gardens may have a more pronounced impact on vitality, life satisfaction, and stress reduction among groups at higher mental health risks, suggesting that digital therapeutic gardens could serve as an appropriate therapeutic intervention for these populations. However, these results were collected from cross-sectional data reflecting the participants’ past experiences, and further controlled studies are required to establish causal relationships.

The advantages and necessity of digital therapeutic gardens identified through this study are as follows. First, digital therapeutic gardens, owing to the characteristics of digital media, are less constrained by environmental factors such as location, time, and weather compared to traditional garden activities. Current nature-based therapy research focuses primarily on the effects of experiences in natural environments (e.g., forests, nature reserves, wetlands, and urban parks) on mood, stress recovery, and mental health (Li et al., 2022; Yang et al., 2023). However, natural environments vary with season and weather, making it difficult to guarantee consistent therapeutic effects. By utilizing digital technology to create virtual environments, elements such as weather and lighting can be controlled to overcome time and space constraints and allow multiple scenes to be presented in a short time, making it possible to explore the effectiveness of therapeutic elements (Li et al., 2022).

Second, digital therapeutic gardens share the usability and convenience of digital healthcare systems. Since the onset of the COVID-19 pandemic, the public has experienced various digital healthcare services, and expectations and demands for these services have expanded beyond simple health monitoring to actively integrate them into disease prevention and treatment (Philippe et al., 2022). Digital therapeutic gardens have been shown to be effective in improving mental health (Kim and Lee, 2023; Lin et al., 2020; Nicholas et al., 2023; Szczepañska-Gieracha et al., 2021). Considering the physical benefits derived from physical activity in nature-based gardens (Park et al., 2016) digital therapeutic gardens with active participation can promote both mental and physical health. Additionally, digital therapeutic gardens could provide the benefits of natural environments to individuals who find it difficult to visit actual nature-based therapy settings (e.g., the elderly, the disabled, or those living in areas with limited transportation), making them efficient and effective digital healthcare tools.

Finally, digital therapeutic gardens are likely to offer advantages in terms of cost-effectiveness. VR-based therapy has been reported to enhance the effectiveness of professional mental health treatments by providing supplementary therapeutic effects with high-cost efficiency and accessibility (Geraets et al., 2021). Given the reported potential effectiveness of therapeutic gardens in alleviating anxiety and depression, digital therapeutic gardens can also be effectively applied to prevent or mitigate mild symptoms of anxiety and depression. The use of natural elements in digital therapeutic gardens may reduce treatment resistance and side effects (Lin et al., 2019).

### 4.2 Practical implications

This study suggests that digital therapeutic gardens can function as a complementary approach for promoting psychological well-being and social connectedness in communities. For healthcare practitioners, digital therapeutic gardens may serve as innovative interventions to enhance mental health care by providing accessible and personalized therapeutic environments. Specifically, digital therapeutic gardens can be applied as emotion regulation strategies to support individuals with clinical levels of depression, while also functioning as a preventive approach for those experiencing stress, depression, anxiety, isolation and loneliness.

Further research is needed to rigorously evaluate the effectiveness of digital therapeutic gardening, but its potential applications across multiple domains deserve careful consideration. For example, urban planners and local governments could incorporate digital gardens into public spaces to promote stress recovery and community engagement in highly urbanized areas. Schools may benefit from integrating digital gardens into learning environments to support students’ attention restoration and emotional regulation. Furthermore, it is worth researching whether digital gardening contributes to the improvement of Nature Quotient (NQ), defined as the “capacity to perceive, process and organize information about ecological interconnections” (Vuong and Nguyen, 2025). If so, digital therapeutic gardens could be utilized for education to cultivate in students a heightened awareness of the critical importance of biodiversity conservation and proactive responses to climate change. From a policy perspective, digital therapeutic gardens may serve as innovative tools to support sustainable and inclusive health strategies. Although empirical validation is still required, these possibilities highlight the value of further exploring the effect of public health and sustainable urban development.

### 4.3 Limitations

Nevertheless, this study has certain limitations. First, this study is limited to clarifying the concept of digital therapeutic garden and relies on cross-sectional surveys. Longitudinal follow-up studies would be required to validate the therapeutic effects of digital gardens. Moreover, future research should examine the therapeutic effects of different types of digital gardens based on our classification, incorporating comprehensive psychological assessments and physiological indicators (e.g., heart rate variability, galvanic skin response) for more robust validation. As the empirical literature on the effectiveness of digital therapeutic gardens expands, future meta-analyses and systematic reviews will be essential for consolidating the evidence base. These approaches will contribute to refining the conceptualization of digital therapeutic gardens and advancing theoretical clarity in this emerging field.

Despite the advantages of digital therapeutic gardens in terms of accessibility, there are still limitations in providing individualized psychological services that consider personal factors such as symptom severity, personality, and age (Borghouts et al., 2021). To overcome this problem, digital therapeutic gardens can be more effective when integrated with face-to-face psychological services (Ehrt-Schaefer et al., 2022). Additionally, for the physical therapeutic effects arising from direct contact with nature (Alvarsson et al., 2010; Gladwell et al., 2012; Li et al., 2009, 2011), digital and traditional therapeutic gardens must be linked. Therefore, for the effective utilization of digital therapeutic gardens, a connection should be established between existing therapeutic gardens and mental health services. For instance, in cases where individuals experience moderate to severe depression, anxiety, or social isolation, offering digital therapeutic gardens can improve accessibility to treatment and ultimately facilitate linkages with community mental health centers, specialized psychological support centers, and medical institutions. Meanwhile, according to the perception survey, the most significant barrier reported to participation in digital therapeutic gardens was the difficulty in using digital devices and technical challenges. Even if time and space constraints are overcome, access to digital devices can still be a barrier. Therefore, it is necessary to create detailed manuals for each program and develop a training program for practitioners to facilitate the easy use of digital devices and applications, regardless of age or region. Finally, developing and applying digital therapeutic gardens tailored to specific populations may be more effective. For instance, a telehealth-based therapeutic garden conducted via videoconference could be more suitable for older adults or individuals with intellectual disabilities who may struggle with app-based platforms. Evidence shows that telehealth-based horticultural therapy reduces dropout rates and helps alleviate stress, depression, and loneliness (Meore et al., 2024), suggesting that telehealth approaches should be expanded to therapeutic gardening.

## 5 Conclusion

This study categorizes the types of digital therapeutic gardens and analyzes the relationship between public perception, participation experience, and mental health variables. The findings indicate that the participants of digital therapeutic reported the greatest improvements in social interaction, suggesting that digital gardening may contribute to enhancing social participation and connectedness. According to further analysis, the experience of digital gardens could be related to reduced stress and increased vitality and life satisfaction. Based on the concept of digital therapeutic gardens proposed in this study, we expect that future research will further investigate its therapeutic effectiveness and that these findings will serve as a foundation for the implementation of digital therapeutic gardens. Also, it is expected that digital therapeutic gardens that overcome the constraints of time and space to achieve mindfulness, connectedness to nature, and social interaction may be a candidate for evidence-based interventions for mental health improvement. These findings highlight the importance of designing digital therapeutic gardens that are accessible, engaging, and capable of promoting social interaction and relaxation for practitioners and garden facilitators. Mental health service providers may consider integrating digital therapeutic gardens into community-based or clinical programs to support psychological well-being and social connectedness. It would be helpful to promote awareness, ensuring accessibility, and provide technical support for digital therapeutic gardens from a policy perspective to overcome barriers to participation and maximize public benefit.

## Data Availability

The raw data supporting the conclusions of this article will be made available by the authors, without undue reservation.

## Supplementary material

Supplementary Figure 1 Study framework.
