# Supplementary figures and images for: The concept of the digital therapeutic garden and its psychological effects
Source: Front Psychol. 2025 Oct 31;16:1534541. doi: 10.3389/fpsyg.2025.1534541 (PMC12615244; doi:10.3389/fpsyg.2025.1534541)

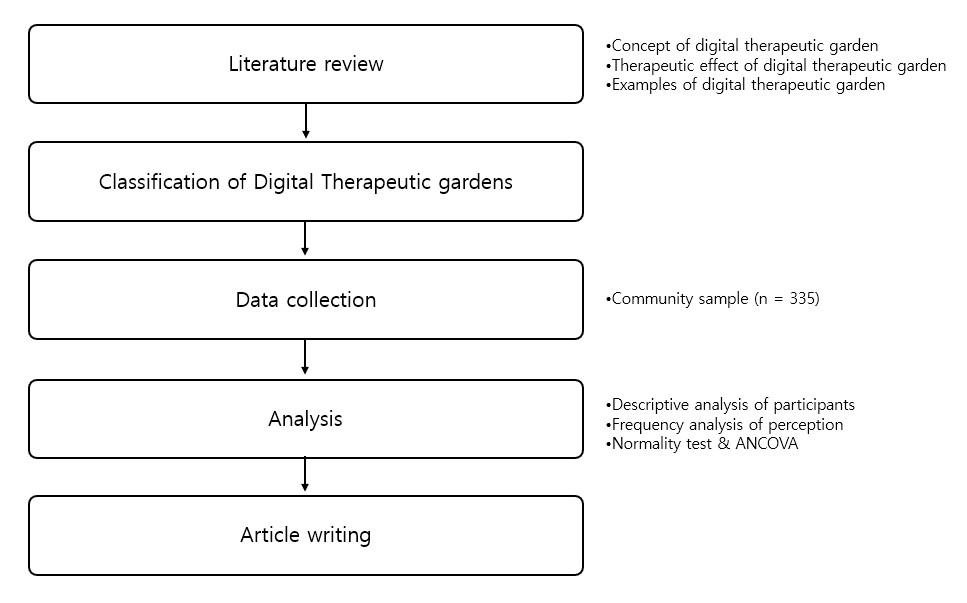

Supplement: Supplementary Figure 1 — Study framework. [file Image_1.PNG]
